# DNA Barcoding Reveals High Cryptic Diversity of the Freshwater Halfbeak Genus *Hemirhamphodon* from Sundaland

**DOI:** 10.1371/journal.pone.0163596

**Published:** 2016-09-22

**Authors:** HongChiun Lim, Muchlisin Zainal Abidin, Chaidir Parlindungan Pulungan, Mark de Bruyn, Siti Azizah Mohd Nor

**Affiliations:** 1 School of Biological Sciences, Universiti Sains Malaysia, Penang, Malaysia; 2 School of Biological Sciences, Faculty of Integrative Sciences and Technology, Quest International University Perak, Ipoh, Perak, Malaysia; 3 Department of Aquaculture, Faculty of Marine and Fisheries, Syiah Kuala University, Banda Aceh, Indonesia; 4 Department of Aquatic Resources Management, Faculty of Fisheries and Marine Science, Riau University, Pekanbaru, Indonesia; 5 School of Biological Sciences, Bangor University, Bangor, United Kingdom; 6 School of Biological, Earth and Environmental Sciences, University of New South Wales, Sydney, Australia; 7 Centre for Marine and Coastal Studies, Universiti Sains Malaysia, Penang, Malaysia; University of Roehampton, UNITED KINGDOM

## Abstract

DNA barcoding of the cytochrome oxidase subunit I (COI) gene was utilized to assess the species diversity of the freshwater halfbeak genus *Hemirhamphodon*. A total of 201 individuals from 46 locations in Peninsular Malaysia, north Borneo (Sarawak) and Sumatra were successfully amplified for 616 base pairs of the COI gene revealing 231 variable and 213 parsimony informative sites. COI gene trees showed that most recognized species form monophyletic clades with high bootstrap support. Pairwise within species comparisons exhibited a wide range of intraspecific diversity from 0.0% to 14.8%, suggesting presence of cryptic diversity. This finding was further supported by barcode gap analysis, ABGD and the constructed COI gene trees. In particular, *H*. *pogonognathus* from Kelantan (northeast Peninsular Malaysia) diverged from the other *H*. *pogonognathus* groups with distances ranging from 7.8 to 11.8%, exceeding the nearest neighbor taxon. High intraspecific diversity was also observed in *H*. *byssus* and *H*. *kuekanthali*, but of a lower magnitude. This study also provides insights into endemism and phylogeographic structuring, and limited support for the Paleo-drainage divergence hypothesis as a driver of speciation in the genus *Hemirhamphodon*.

## Introduction

Biological diversity is believed to be entering an era of mass extinction where it is disappearing worldwide at unprecedented rates [1]. Thus, precise taxonomic delineation of species is crucial in the context of biodiversity conservation [1]. Traditionally, species description and identification are based on morphological traits, however, the morphological approach alone has intrinsic limitations. Phenotypic plasticity and genotypic variation may mask the morphological characters used in species descriptions, leading to misdiagnoses. Furthermore, cryptic diversity cannot be easily detected, and variable life stage morphologies and sexual dimorphism may add to the confusion, thus requiring high dependence on experts, often a time consuming enterprise [2]. Consequently, molecular methods are proposed to contribute to a new ‘integrative taxonomy’ approach, which can enhance speed and accuracy in species discovery [1,3].

The DNA barcoding concept was first proposed by Hebert *et al*. [4], based on an approximately 655 base pair (bp) fragment of cytochrome c oxidase subunit I (COI), which has since been adopted as the gold standard for global bioidentification to differentiate among animal species. This system proposed that intraspecific COI divergence would be less than interspecific divergence. Thus, the delimitation of species in DNA barcoding is based on this so-called “barcoding-gap”. The evolutionary rate, absence of indels, and limited occurrence of recombination are the primary reasons why COI was chosen as the DNA barcoding gene, in addition to the existence of robust universal primer sets that can amplify across diverse taxonomic groups [5]. Rapid identification of potentially unidentified species in global biodiversity assessment and conservation, such as cryptic species, juveniles and organisms with ambiguous morphological characters, is one of the main goals of DNA barcoding.

Species identification of freshwater and marine fishes through the utilisation of the DNA barcoding method have resulted in a greater than 90% success rate in species discrimination [5–11]. The inconsistencies from expectations as determined by other approaches might be attributable to several factors such as maintenance of ancestral polymorphism, recent divergence among lineages and introgressive hybridization [11,12]. Since the development of DNA barcoding, there has been extensive documentation of species discovery and cryptic species revelations from DNA barcoding data for both freshwater and marine fishes [13–17].

The freshwater fish genus *Hemirhamphodon* [18] has received considerable global interest among scientists [19–21] working in the biodiversity-rich region of Sundaland in Southeast (SE) Asia. Its wide distribution and endemism within Sundaland makes it suitable for phylogenetic, population and phylogeographic studies, and even the potential discovery of new species. *Hemirhamphodon* belongs to the family Hemiramphidae, subfamily Zenarchopterinae [22]. Members of the family are commonly known as halfbeaks due to their characteristic long needle-like lower jaw (beak), but shorter and triangular upper jaw, which is less than half the length of the lower jaw. It inhabits small freshwater rivers and streams and is distributed only in specific areas of Sundaland (southern Thailand, Peninsular Malaysia, Sumatra, Java and Borneo) [23]. *Hemirhamphodon* is unique from other halfbeak genera in having anteriorly directed teeth along the entire length of the lower jaw [23], and pleural ribs originating from the second vertebra instead of the third [21].

Anderson and Collette [23] described a total of six species based on the number of vertebrae, dorsal fins, anal fins, and the pattern of melanophores and pigments on the dorsal fin. The six species are *H*. *phaiosoma* [24], *H*. *pogonognathus* [25], *H*. *kuekenthali* [26], *H*. *chrysopunctatus* [27], *H*. *kapuasensis* [23] and *H*. *tengah* [23]. More recently, three new species from Borneo are currently assigned as *H*. *byssus*, *H*. *sesamun* and *H*. *kecil* [21]. According to Roberts [28], *H*. *pogonognathus* is the most widespread species in this genus. Its distribution covers almost all of the river basins in Sundaland except northern Borneo (Sabah) and even extends out of Sundaland to the Moluccas (Halmahera) [23] (Anderson and Collette, 1991). Observations by Roberts [28], Brembach [27], Wickman [29] and Hartl [30], revealed that *H*. *pogonognathus* coloration shows some differentiation amongst some localities. Tan and Lim [21] observed that *H*. *kuekenthali* is only found in the northern region of Sarawak and is believed to be endemic to the river basins in northern region of Sarawak. On the other hand, the newly assigned species with an apparently allopatric distribution, *H*. *byssus*, is believed to be endemic to the river basins in Sarawak mainly inhabiting the southern region of Sarawak. Although eight species (*H*. *byssus*, *H*. *kuekenthali*, *H*. *kapuasensis*, *H*. *tengah*, *H*. *chrysopunctatus*, *H*. *phaiosoma*, *H*. *sesamum* and *H*. *kecil*) occur in Borneo, *H*. *byssus* and *H*. *kuekenthali*, which are endemic to Sarawak, are geographically separated from the other six species by mountain ranges traversing the island of Borneo. *Hemirhamphodon sesamum* that has been discovered in eastward flowing lowland coastal basins of South Kalimantan is most closely related to *H*. *kuekenthali* in terms of external morphology [21]. *Hemirhamphodon kecil* (“kecil” means small in the Malay/Indonesian language) has a smaller adult size compared to its closest congener, *H*. *pogonognathus* and is found in the lower Mahakam basin in east Kalimantan. *Hemirhamphodon kapuasensis* is endemic to the Kapuas river basin in Kalimantan Barat. The most morphologically distinct species is *H*. *phaiosoma* with a relatively higher number of dorsal-fin rays. However, based on reproductive behavior, *H*. *tengah* seems to be the most distinct species. As noted above, the *Hemirhamphodon* genus are livebearers or viviparous, but *H*. *tengah* has been observed to lay fertilized eggs [31–33]. This, undoubtedly, is a key factor that has generated much interest in the scientific biodiversity community.

This study focused on the preliminary assessment of the species diversity within the genus *Hemirhamphodon* through DNA barcoding. Based on the findings of Anderson and Collette [23] and the revision by Tan and Lim [21], we hypothesise that there is potentially high cryptic diversity in this genus due to its broad distribution and locale-specific polymorphisms.

## Methodology

### Ethic Statement

This study was carried out in accordance with the recommendations and approval by the Universiti Sains Malaysia Animal Ethics Committee. No protected species were collected in this study. Permission was granted from the State Forestry Department for field sampling in Peninsular Malaysia and Sarawak. Samples from Sumatra were obtained from collaborators from Syiah Kuala University and Riau University in Sumatra.

### Sample collection, preservation and DNA extraction

**Fig 1:**
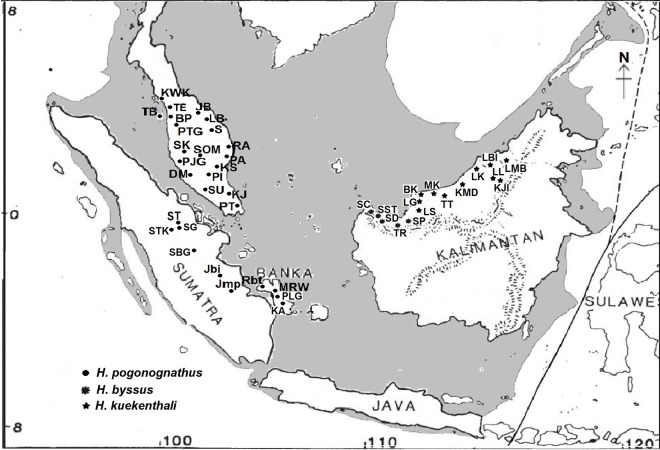
Sampling locations of *H*. *pogonognathus*, *H*. *byssus* and *H*. *kuekenthali* (Modified from Anderson and Collette, 1991), for location abbreviation see Table 1.

Collection of *H*. *pogonognathus*, *H*. *kuekenthali* and *H*. *byssus* species samples were conducted in Peninsular Malaysia, Sarawak and Sumatra as shown in Fig 1 and Table 1. The information on geographical locations and voucher specimens is shown in S1 Table. Specimens were euthanised with *Transmore* (NIKA Trading Co.), a commercial fish stabilizer commonly used in aquatic trading in Malaysia before the fin clips were excised from the pectoral fin of the specimens and stored in 95% ethanol for DNA extraction. Specimens were subsequently formalin fixed and preserved in 70% ethanol as vouchers. Voucher specimens were identified based on taxonomic keys by Anderson and Collette [23] and Tan and Lim [21]. Images of voucher specimens were captured by digital camera prior to being placed in 95% ethanol for long-term storage. Voucher specimens for each COI barcode were deposited at the Museum of Biodiversity, Universiti Sains Malaysia. DNA extractions were conducted using the high-salt DNA extraction protocol [34] and then stored at -20°C until required.

**Table 1:** Species name, locations, code, sample size (n), regional locations within Peninsular Malaysia, Sarawak (Borneo) and Sumatra; and the newly assigned groups of *Hemirhamphodon* species.

Species	Locations	Code	n	Present region of locations	Newly assigned groups
*Hemirhampodon pogonognathus*	Kampung Wang Kelian	KWK	5	Northwest-Peninsular	Main
*H*. *pogonognathus*	Sungai Teroi	TE	5	Northwest-Peninsular	Main
*H*. *pogonognathus*	Teluk Bahang	TB	5	Northwest-Peninsular	Main
*H*. *pogonognathus*	Bukit Pancor	BP	5	Northwest-Peninsular	Main
*H*. *pogonognathus*	Pondok Tanjung	PTG	5	Northwest-Peninsular	Main
*H*. *pogonognathus*	Sungkai	SK	5	Northwest-Peninsular	Main
*H*. *pogonognathus*	Sungai Panjang	PJG	5	West-Peninsular	Main
*H*. *pogonognathus*	Damansara	DM	5	West-Peninsular	Main
*H*. *pogonognathus*	Serting Ulu	SU	5	Central-Peninsular	Main
*H*. *pogonognathus*	Kampung Som	SOM	5	Central-Peninsular	Main
*H*. *pogonognathus*	Kampung Salong	KS	5	Central-Peninsular	Main
*H*. *pogonognathus*	Pos Iskandar	PI	5	Central-Peninsular	Main
*H*. *pogonognathus*	Jeram Pasu	JP	5	Northeast-Peninsular	Kelantan
*H*. *pogonognathus*	Lata Belatan	LB	5	Northeast-Peninsular	Kelantan
*H*. *pogonognathus*	Sekayu	S	5	Northeast-Peninsular	Main
*H*. *pogonognathus*	Rantau Abang	RA	5	East-Peninsular	Main
*H*. *pogonognathus*	Padang Ah Hong	PA	5	East-Peninsular	Main
*H*. *pogonognathus*	Panti	PT	5	Southeast-Peninsular	Main
*H*. *pogonognathus*	Kahang Jemaluang	KJ	5	Southeast-Peninsular	Main
*H*. *pogonognathus*	Sungai Tarai	ST	3	Central-east-Sumatra	Main
*H*. *pogonognathus*	Sungai Gerigeng	SG	3	Central-east-Sumatra	Main
*H*. *pogonognathus*	Sungai Timek	STK	3	Central-east-Sumatra	Main
*H*. *pogonognathus*	Sungai Baung	SBG	3	Central-east-Sumatra	Southern Sumatra
*H*. *pogonognathus*	Jambi	JBI	3	Southeast- Sumatra	Southern Sumatra
*H*. *pogonognathus*	Jambi Palembang	Jmp	3	Southeast- Sumatra	Southern Sumatra
*H*. *pogonognathus*	Rambat	Rbt	3	North-Bangka Island, Southeast-Sumatra	Southern Sumatra
*H*. *pogonognathus*	Merawang	MRW	3	Central-Bangka Island, Southeast-Sumatra	Southern Sumatra
*H*. *pogonognathus*	Petaling	PLG	3	Central-Bangka Island, Southeast-Sumatra	Southern Sumatra
*H*. *pogonognathus*	Koba	KA	2	South-Bangka Island, Southeast-Sumatra	Southern Sumatra
*H*. *byssus*	Kampung Semunin Cina	SC	5	Southern-Sarawak, Borneo	Southern
*H*. *byssus*	Sungai Stuum Toman	SST	5	Southern-Sarawak, Borneo	Southern
*H*. *byssus*	Sungai Duyoh	SD	4	Southern-Sarawak, Borneo	Southern
*H*. *byssus*	Tapang Rumput	TR	4	Southern-Sarawak, Borneo	Central
*H*. *byssus*	Sungai Paku	SP	5	Southern-Sarawak, Borneo	Central
*H*. *kuekenthali*	Nangan Lassi	LS	5	Northern-Sarawak, Borneo	Central
*H*. *kuekenthali*	Nangan Lanang	LG	4	Northern-Sarawak, Borneo	Central
*H*. *kuekenthali*	Bukit Kemunyang	BK	5	Northern-Sarawak, Borneo	Central
*H*. *kuekenthali*	Mukah	MK	5	Northern-Sarawak, Borneo	Central
*H*. *kuekenthali*	Tatau	TT	5	Northern-Sarawak, Borneo	Central
*H*. *kuekenthali*	Sungai Kemenda	KMD	5	Northern-Sarawak, Borneo	Northern
*H*. *kuekenthali*	Sungai Liku	LK	3	Northern-Sarawak, Borneo	Northern
*H*. *kuekenthali*	Long Lama	LL	5	Northern-Sarawak, Borneo	Northern
*H*. *kuekenthali*	Sunagi Kejin	KJI	5	Northern-Sarawak, Borneo	Northern
*H*. *kuekenthali*	Labi-Linei	LBI	4	Northern-Sarawak, Borneo	Northern
*H*. *kuekenthali*	Limbang	LMB	5	Northern-Sarawak, Borneo	Northern

### Gene amplification and sequencing

A partial segment of the COI gene of 3 to 5 individuals per location (except only 2 individuals from Koba) were PCR amplified using BIO-RAD T100 Thermal Cycler (BioRad Laboratories Inc., USA) with primers Fish F2 and Fish R2 [6]. The PCR conditions were conducted as described in Ward *et al*. [6]. The PCR products were electrophoresed on 1.5% agarose gels for band characterization and purified (PCR Clean-up System, Promega, Madison, WI, USA). Sequencing was conducted by a service provider (First Base Laboratories Sdn. Bhd., Malaysia) using an ABI3730XL Genetic Analyzer (Applied Biosystems, Foster City, CA, USA).

### Data Analysis

All sequences were edited using MEGA v6.0 [35]. Multiple alignments were evaluated using MUSCLE [36] that is integrated within MEGA. Minor adjustments were then made by eye to manually remove any false homologies. The pairwise comparison matrices were constructed using the Kimura 2 Parameter (K2P) model as it is most widely used in COI barcoding studies [2,37]. To check the presence of a “barcode gap” in our dataset, the maximum intraspecific divergences against the minimum nearest-neighbour divergences was plotted.

The Automatic Barcode Gap Discovery (ABGD) species delineation tool on a web interface (http://wwwabi.snv.jussieu.fr/public/abgd/abgdweb.html) with default settings for the K2P distance matrix was employed [38] to determine the number of operational taxonomic units (OTUs) based on pairwise sequence distances between individuals within the dataset. A Neighbour-Joining (NJ) COI gene tree was constructed using K2P models with the bootstrap procedure [39] of 10000 pseudoreplicates. A Bayesian Inference (BI) COI gene tree was included to examine any likelihood of different positioning of OTUs. The BI tree was constructed using MrBayes [40] with HKY+G+I model (optimal substitution model under Bayesian Information Criterion using ModelTest in MEGA) for 1000 pseudoreplicates. The length of the MCMC chain was 5 million with a sample frequency of 500.

## Results

A total of 201 individuals were successfully PCR amplified for the COI gene consisting of three (morphologically) recognized species. All sequences have been deposited in GenBank (accession number: KM405651 –KM405787 and KX216532 –KX216595) and are available on BOLD public datasets DS-HMRD. Several additional species and two out-group sequences totaling 112 individuals from GenBank (Table 2) were also included in the analyses. Three other documented species, *H*. *kapuasensis*, *H*. *kecil* and *H*. *sesamun* were not included in the analyses as no specimens were obtained and no GenBank sequences were available. The final COI gene segment consisted of 616bp with average nucleotide composition of A = 24%, T = 35%, C = 25% and G = 16%, 231 variable sites, of which 213 were parsimony informative. No insertions, deletions or stop codons after translation were found, indicating that the amplified sequences constitute functional mitochondrial COI sequences and did not harbour any nuclear mitochondrial pseudogenes (numts).

**Table 2:** Sequences of the COI gene of *Hemirhamphondon* species obtained from GenBank.

Species	Sequence label	GenBank Accession
*Hemirhamphodon pogonognathus*	H_7140	JQ430593.1
*H*. *kuekenthali* *(H. *byssus)*	H_7136	JQ430639.1
*H*. *kuekenthali*	H_7138	JQ430651.1
*H*. *phaiosoma*	H_7135	JQ430652.1
*H*. *tengah*	H_7129	JQ430653.1
*H*. *tengah*	H_7142	JQ430654.1
*H*. *chrysopunctatus*	H_7141	JQ430655.1
*H*. *sp*.	H_7125	JQ430557.1
*H*. *sp*.	H_7127	JQ430054.1
*H*. *sp*.	H_7126	JQ430561.1
*H*. *sp*.	H_SGK1	JQ430601.1
*H*. *sp*.	H_Pek1 –H_Pek10	JQ430558.1, JQ430560.1, JQ430563.1—JQ430565.1, JQ430567.1, JQ430571.1, JQ430577.1, JQ430582.1, JQ430586.1
*H*. *sp*.	H_Pen1 –H_Pen11	JQ430573.1, JQ430575.1, JQ430579.1, JQ430584.1, JQ430594.1, JQ430599.1, JQ430602.1, JQ430603.1, JQ430605.1, JQ430607.1, JQ430609.1
*H*. *sp*.	H_Sel2—H_Sel3	JQ430559.1, JQ430562.1
*H*. *sp*.	H_Jam1 –H_Jam10	JQ430546.1—JQ430553.1, JQ430555.1—JQ430556.1
*H*. *sp*.	H_LR_5250—H_LR5260	JQ430569.1, JQ430572.1, JQ430576.1, JQ430578.1, JQ430580.1, JQ430583.1, JQ430585.1, JQ430600.1, JQ430608.1, JQ430610.1, JQ430611.1
*H*. *sp*.	H_LR_5317—H_LR5326	JQ430612.1—JQ430621.1
*H*. *sp*.	H_LR_5422—H_LR5425	JQ430588.1, JQ430589.1, JQ430591.1, JQ430606.1
*H*. *sp*.	H_LR_5445—H_LR5449	JQ430574.1, JQ430590.1, JQ430592.1, JQ430595.1, JQ430604.1
*H*. *sp*.	H_LR_5452—H_LR5459	JQ430566.1, JQ430568.1, JQ430570.1, JQ430581.1, JQ430587.1, JQ430596.1, JQ430597.1, JQ430598.1
*H*. *sp*.	H_LR_6979—H_LR6980d	JQ430634.1—JQ430638.1
*H*. *sp*.	H_LR_6993—H_LR7003	JQ430640.1—JQ430650.1
*H*. *sp*.	H_Sar1- H_Sar12	JQ430622.1—JQ430633.1
*Dermogenys sp*.	D_Pen4	JQ430526.1
*Dermogenys sp*.	D_Pen7	JQ430524.1

*New species name according to the latest species revision by Tan and Lim, 2013.

**Fig 2:**
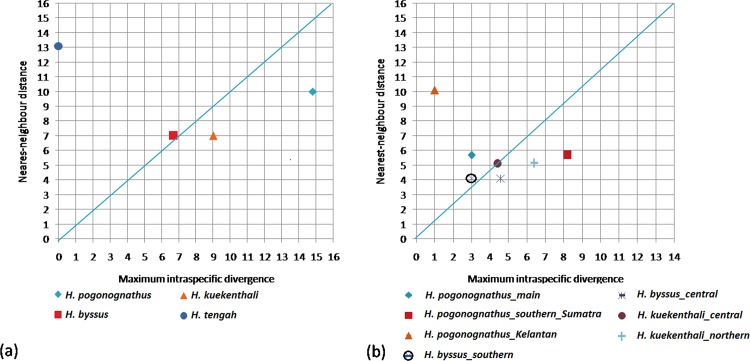
Maximum intraspecific divergence compared with nearest-neighbour distance for *Hemirhamphodon* species excluding two singleton species. Diagonal line represents 1:1 line to separate “barcode gap” presence and absence area; (a) Four initial presumed morphological species; (b) Newly assigned *Hemirhamphodon* species grouping.

The summary values of genetic divergence across taxonomic level based on K2P are shown in Table 3. The mean genetic divergence within species was 2.5% while the within genus divergence was three times greater at 8.4%. Pairwise comparisons of interspecific divergence among six species (Table 4) ranged from 8.7% (*H*. *byssus* vs *H*. *kuekenthali*) to 20.1% (*H*. *phaiosoma* vs *H*. *chrysopunctatus—*both from GenBank sequences) with a mean of 15.2%. Intraspecific divergence (S2 Table) revealed considerable heterogeneity ranging from low to deep divergence for *H*. *pogonognathus* (0% to 14.8%), *H*. *byssus* (0% to 6.7%) and *H*. *kuekenthali* (0% to 7.0%). A barcode Gap analysis (Fig 2) with two singleton species (*H*. *phaiosoma* and *H*. *chrysopunctatus*) excluded showed that a barcode gap was present in *H*. *tengah* and *H*. *byssus* while no barcode gap was present in *H*. *pogonognathus* and *H*. *kuekenthali*.

**Table 3:** Sample size (n), mean values, ranges of genetic divergences based on K2P across taxonomic levels from 311 sequences of the genus *Hemirhamphodon*.

Taxon	*n*	Min	Max	Mean
Within Species	*4	0	4.8	2.5
Within Genus	311	0	20	8.4

*Only four species analysed as the other two species were represented by a single individual.

**Table 4:** Pairwise comparisons of the COI gene based on K2P distance among six presumed (morphologically identified) *Hemirhamphodon* species.

		1	2	3	4	5	6	7
1	*H_pogonognathus*	0.045						
2	*H_byssus*	0.126	0.030					
3	*H_kuekenthali*	0.120	0.087	0.048				
4	*H_phaiosoma*	0.144	0.143	0.148	n/c			
5	*H_chrysopunctatus*	0.171	0.186	0.178	0.201	n/c		
6	*H_tengah*	0.150	0.149	0.145	0.177	0.161	0.000	
7	*Outgroup_Dermogenys*	0.186	0.215	0.206	0.201	0.208	0.150	0.000

n/c = no calculation due to single sample.

**Fig 3:**
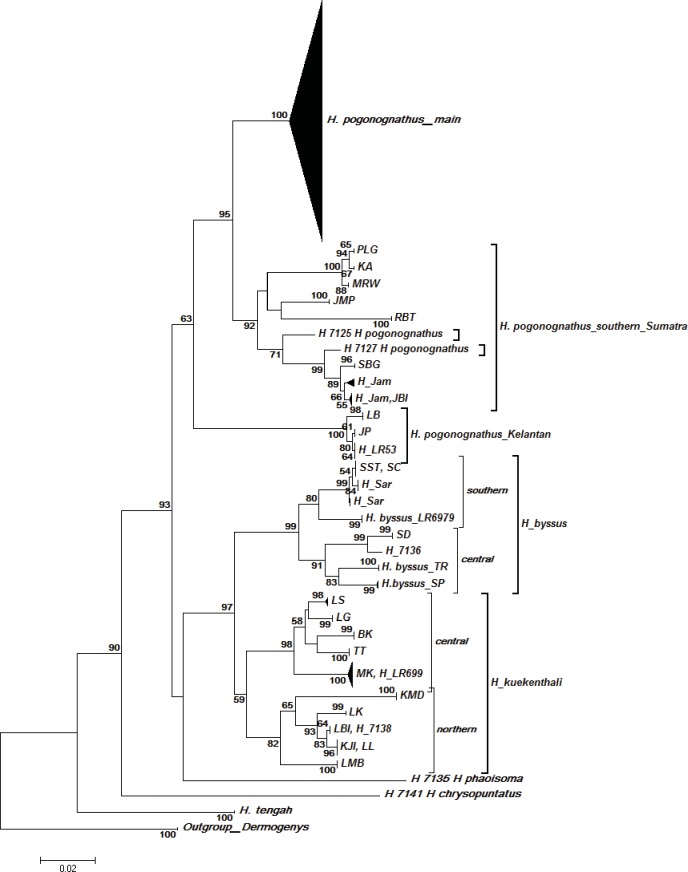
Neighbor-Joining COI gene tree among *Hemirhamphodon* species generated through K2P. Values at nodes represent bootstrap confidence levels (10000 replicates). A *Dermogenys* species was employed as an outgroup.

**Fig 4:**
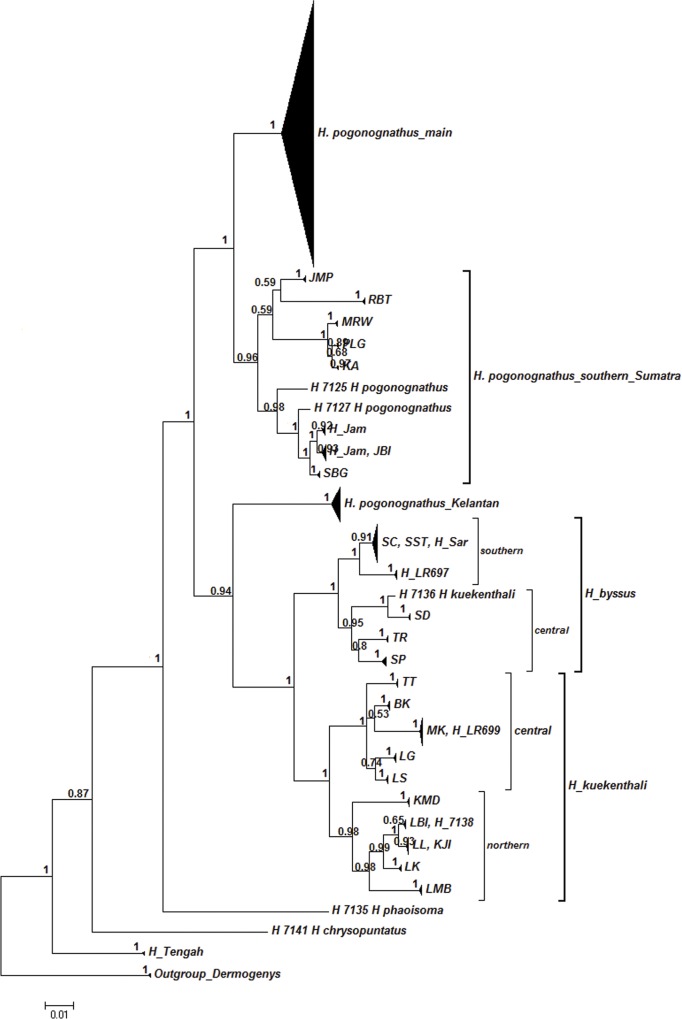
Bayesian Inference COI gene tree generated through HKY+G+I. Value at nodes represents the Bayesian posterior probability. A *Dermogenys* species was employed as an outgroup.

The constructed NJ (Fig 3) COI gene tree consisted of nine OTUs including one outgroup. Most individuals formed monophyletic species clusters consistent with their morphological identifications. However, *H*. *pogonognathus* was split into three distinct clusters with one major cluster of *H*. *pogonognathus* from Peninsular Malaysia and central Sumatra, a second cluster of a southern Sumatran group, and a third discrete Kelantan (JP, LB and H_LR53) cluster. Further inspection also revealed that population divergence patterns occurred in *H*. *byssus* and *H*. *kuekenthali*. *H*. *byssus* of the southern group (SC and SST) formed one cluster while *H*. *byssus* from the central group (SD, SP and TR) formed another cluster. For *H*. *kuekenthali*, the northern group (KMD, LK, KJI, LL, LBI and LMB) formed one cluster and the central group (LS, LG, BK, MK and TT) formed another cluster. The BI COI gene tree (Fig 4) showed nearly the same OTU clusters except for *H*. *pogonognathus* from Kelantan, where it branched as a sister clade of *H*. *kuekenthali*, instead of the main *H*. *pogonognathus* group as shown in the NJ tree.

**Fig 5:**
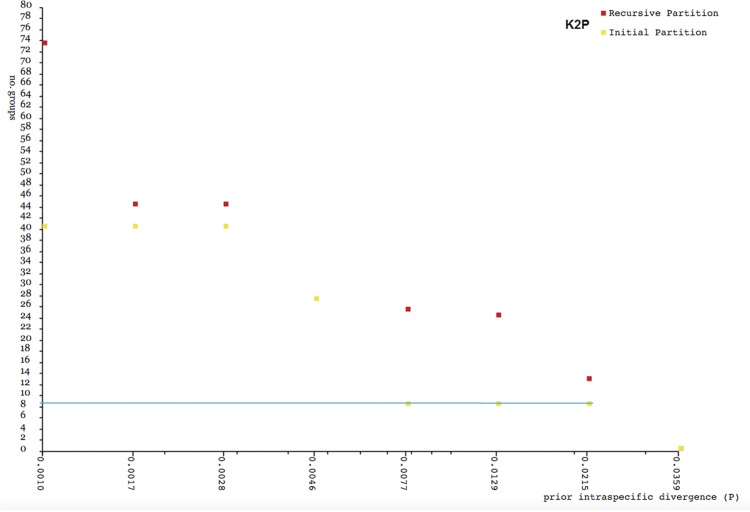
The number of genetically distinct OTUs according to the prior intraspecific divergence value generated by ABGD based on K2P.

The number of OTUs generated by ABGD based on K2P varied from 1 to 74 (Fig 5). The initial partition at a prior intraspecific divergence (*P*) (*P* = 0.0077–0.0359) produced 9 OTUs, and was in concordance with the NJ and BI trees. The additional OTU identified by ABGD was *H*. *pogonognathus* from Rambat.

Pairwise comparisons (Table 5) were computed for the newly assigned groupings according to the constructed NJ COI gene tree and the ABGD results. As expected, intraspecific divergence exhibited a decreased value with a mean of 1.5% ranging from 0% to 4.3%. However, the intraspecific divergence for *H*. *pogonognathus* (southern Sumatra) remained high (4.3%). Pairwise divergence between groups for *H*. *byssus* (southern *vs* central) and *H*. *kuekenthali* (northern *vs* central) were 4.9% and 6.9% respectively. These values might be considered high but did not exceed the minimum nearest-neighbour interspecific divergence (7.9%). On the other hand, pairwise divergence among the three *H*. *pogonongnathus* groups ranged from 7.8% to 11.8%, where divergences of the Kelantan group from other populations exceeded the minimum interspecific divergence value. A re-analysis of the barcode gap was conducted (Fig 2) for the new grouping. Again, the results revealed absence of a barcode gap in *H*. *pogonognathus* from southern Sumatra, *H*. *kuekenthali* from northern and *H*. *byssus* from central.

**Table 5:** Pairwise comparison of the COI gene based on K2P distance among newly assigned *Hemirhamphodon* groupings.

		1	2	3	4	5	6	7	8	9	10	11
1	*H_pogonognathus_main*	0.011										
2	*H_pogonognathus_southern_Sumatra*	0.078	0.043									
3	*H_pogonognathus_Kelantan*	0.107	0.118	0.004								
4	*H_byssus_southern*	0.123	0.124	0.124	0.010							
5	*H_byssus_central*	0.134	0.133	0.120	0.049	0.029						
6	*H_kuekenthali_central*	0.119	0.121	0.124	0.090	0.095	0.025					
7	*H_kuekenthali_northern*	0.120	0.121	0.120	0.079	0.087	0.069	0.030				
8	*H_phaiosoma*	0.141	0.147	0.163	0.138	0.153	0.147	0.148	n/c			
9	*H_chrysopunctatus*	0.168	0.178	0.178	0.193	0.174	0.174	0.182	0.201	n/c		
10	*H_tengah*	0.149	0.149	0.159	0.147	0.154	0.147	0.141	0.177	0.161	0.000	
11	*Outgroup_Dermogenys*	0.181	0.201	0.201	0.214	0.216	0.208	0.203	0.201	0.208	0.150	0.000

n//c = no calculation due to single sample.

**Fig 6:**
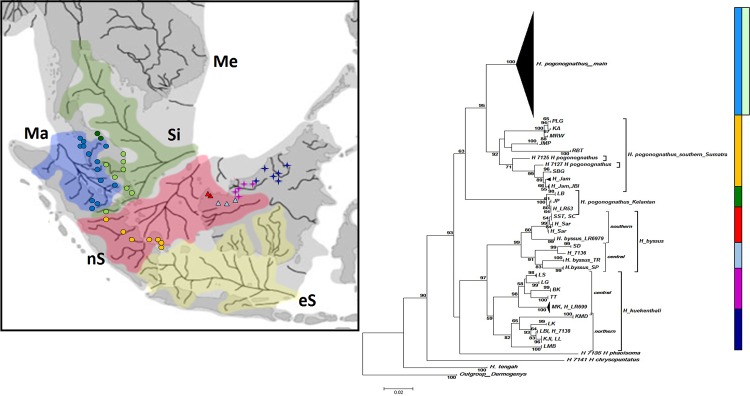
Sampling locations according to Paleo-drainage systems mapped with NJ COI gene tree. Ma = Malacca, Si = Siam, nS = North Sunda, eS = East Sunda, Me = Mekong. *H*. *pogonognathus* (shaded circle), *H*. *byssus* (shaded triangle) and *H*. *kuekenthali* (shaded four-pointed star). Colours of shapes and bars are identical in representing new assigned grouping. The embedded map was reprinted/modified from Voris, 2000 under a CC BY license, with permission from Field Museum of Natural History, Chicago, original copyright 2000.

The results also revealed that members of the *Hemirhamphodon* genus appear to be allopatrically distributed. To determine whether the high intraspecific divergence was influenced by Paleo-drainage systems in Sundaland as discussed in de Bruyn *et al*. [41], the NJ COI gene tree clusterings were mapped against the Paleo-drainages (Fig 6) as suggested by Voris [42]. The mapping results revealed that only the divergence of *H*. *pogonognathus* from southern Sumatra is consistent with the Paleo-drainage (north Sunda) hypothesis. Samples from Malacca and Siam Paleo-drainages formed a single mixed group instead of two groups. Although there is no record of a Paleo-drainage for north Borneo (Sarawak), the divergence of *H*. *byssus* and *H*. *kuekenthali* seem likely to be congruent with a presently unknown barrier.

## Discussion

The DNA barcoding approach is now widely recognized as an efficient tool to facilitate rapid identification of unidentified or unknown taxa through a DNA barcode reference library and also in assessment for conservation purposes, including of cryptic and microscopic organisms, particularly those with morphologically ambiguous characters [1,4]. In this study, DNA barcode analysis was used in an attempt to assess cryptic diversity in the genus *Hemirhamphodon*. The analysis also permitted insights into the influence of Paleo-drainage systems of Sundaland in driving species diversity.

The high levels of intraspecific divergence in *H*. *pogonognathus*, *H*. *byssus* and *H*. *kuekenthali* suggest that this genus exhibits high cryptic diversity. Several studies have reported the same phenomenon, for instance of the fighting fish *Betta* [43], flathead fishes [11] and fishes from Nujiang River [44], which revealed very high species diversity in the absence of apparent morphological differences. The constructed COI gene trees were generally consistent with the current morphological delimitation of *Hemirhamphodon* species, although several species were only represented by a single sequence here. However, the clustering of *H*. *pogonognathus* split into three well-supported clusters with high levels of divergence, except *H*. *pogononathus* from Kelantan (bootstrap value of 59). This result further supports the existence of cryptic diversity within the *H*. *pogonognathus* group. Given its broad distribution in a biodiversity hotspot and the recent documentation of species discovery in *Hemirhamphodon* [21], this is therefore not surprising. This pattern could be interpreted as any of these: a recent speciation event, interspecies hybridization, or as (morphological) misidentification [45–46]. When hybridization occurs, the divergent sequence will cluster with the clade of one of the hybridizing species. Conversely, for cryptic species, a new divergent clade will be apparent that is different from that of any currently recognized species [11]. Our results clearly exhibit the split of different clusters indicating the probable presence of cryptic diversity.

Multiple lineages generated through tree construction demonstrated the potential occurrence of sources from different drainages. The mapping of Sundaland Paleo-drainage systems against the NJ COI gene tree revealed that Paleo-drainages also likely played a role in the high intraspecific divergence values recovered here. This was evident in the *H*. *pogonognathus* southern Sumatran group which follows the north Sunda Paleo-drainage, diverging from the main *H*. *pogononathus* group (combined Malacca and Siam Paleo-drainages) as shown in Fig 6. Additionally, the NJ COI gene tree also shows further splits within *H*. *byssus* and *H*. *kuekenthali* into multiple lineages. However, these bifurcations did not split out from the currently recognized species to form a new cluster as in *H*. *pogonognathus*. de Bruyn *et al*. [41] found little evidence for Paleo-drainage systems driving divergence in *Hemirhamphodon*, but strong support for this mechanism influencing diversity in the halfbeak genus *Dermogenys*. They postulated that life history strategies (*Hemirhamphodon* = forest stream specialists; *Dermogenys* = brackish water generalists) could have been an important determinant of ability to migrate via these vast Pleistocene paleo-river systems, before subsequent allopatric splitting as sea-levels fell and paleo-systems waned. Although the mapping result demonstrated no obvious Paleo-drainage assigned for north Borneo (Sarawak), the different lineages of *H*. *byssus* and *H*. *kuekenthali* seem very likely to be geographically restricted lineages resulting from unidentified barriers. On the other hand, these divergences could also be associated with ecosystem-dependent adaptive radiation. In addition, the Rajang River seems to have acted as an effective barrier leading to endemism of *H*. *byssus* and *H*. *kuekenthali* with *H*. *byssus* only found in the south of the Rajang basin and *H*. *kuekenthali* in the north.

The tree topology showing several distinct clusters within certain species coupled with high levels of intraspecific diversity indicated probable occurrence of cryptic species [44]. High genetic divergence within nominal species can be interpreted as misidentification, or more importantly as cryptic or unrecognized speciation events [11,12,47]. There are several criteria proposed for species delineation based on the DNA barcoding approach. Hebert *et al*. [48] proposed the ‘10X rule’ as an indicator of cryptic speciation. On the other hand, Ward *et al*. [49], who analysed barcode data from about 1000 fish species, showed that individuals were much more likely to be congeneric than conspecific at a level of 2% distance or greater. Another criterion is the use of the barcode gap, which is the distance or gap between the maximum intraspecific and minimum interspecific distances [38,48, 50–52].

Barcode gap analysis for our dataset for both initial groupings and new groupings (Fig 2) revealed that no barcode gap was present in *H*. *pogonognathus*, *H*. *kuekenthali* and *H*. *byssus*, which indicated the probable existence of more than one species within each of these taxa. In addition, the ABGD method generated 9 OTUs, which is nearly concordant with the COI gene trees. The *H*. *pogonognathus* complex formed three OTUs, which potentially implies detectable intraspecific diversity. Thus, referring to the results of the genetic distance, COI gene trees and barcode gap analyses, the existence of high cryptic diversity among our dataset is apparent.

The three genetic lineages across *H*. *pogonognathus* most likely represent species-level taxa, suggesting that *H*. *pogonognathus* consists of at least two distinct species. *Hemirhamphodon pogonognathus* was the most widely distributed species and showed high intraspecific divergence values up to 14.8% with a mean of 4.5%, separated by three geographical splits (Fig 6). Further re-grouping revealed that the *H*. *pogonognathus* complex exhibited deep divergence among the three *H*. *pogonongnathus* groups, ranging from 7.8% to 11.8% (Table 4), where divergences of the Kelantan group from the other two groups exceeded the minimum interspecific divergence (7.9%). No obvious morphological characters were found to distinguish them into different species even though colour differentiation was observed among some localities. In fact, the genetically distant Kelantan group shared the same colour pattern with the main group. In addition, the Kelantan group was separated from the main cluster to form its own clade in the COI gene trees, further supporting the probable existence of cryptic species in the *H*. *pogonognathus* group. Thus, we propose that *H*. *pogonognathus* from Kelantan has the potential of being a new species record.

The six morphological species included in this study generated 9 OTUs. This DNA barcoding study shows the high potential in cryptic species assessment particularly within ‘hyperdiverse’ SE Asia. The species status for *H*. *byssus* and *H*. *kuekenthali* remains unclear, and each could be considered as a species complex. Based on this study, the genus of *Hemirhamphodon* is proposed to consist of at least 10 species namely *H*. *pogonognathus*, *H*. *kuekenthali*, *H*. *byssus*, *H*. *phaiosoma*, *H*. *chrysopunctatus*, *H*. *tengah*, *H*. *kapuasensis*, *H*. *sesamun*, *H*. *kecil* (the last three species not included in this analysis) and the newly proposed *H*. *pogonognathus* sp. of Kelantan. Nevertheless, further investigations with combined molecular and morphological approaches, and also population level analyses with larger sample sizes are needed to clarify *Hemirhamphodon* taxonomy.

## Conclusions

In conclusion, the present study exhibited the power of DNA barcoding in species diversity assessment. In addition, the findings highlight the high cryptic diversity of this genus, which is in agreement with our initial hypothesis. However, a more integrated study including molecular and morphological approaches needs to be conducted to resolve the issue of the identification of paraphyletic or species complexes of several *Hemirhamphodon* species. Lastly, more studies of freshwater fishes with a range of distributional, life history and other datasets (genetic, phenotypic and geographical) incorporating multiple research tools need to be conducted in order to have a better understanding of the drivers and maintenance of biodiversity within the SE Asian region.

## Supporting Information

S1 Table Geographical coordinates of sampling locations and voucher specimen repository. (XLSX)

S2 Table Pairwise comparisons of the COI gene based on K2P distance among sampling locations of *Hemirhamphodon* species. (XLSX)
